# Supplementary material for: Molecular characterization of Chlamydomonas reinhardtii telomeres and telomerase mutants
Source: Life Sci Alliance. 2019 Jun 3;2(3):e201900315. doi: 10.26508/lsa.201900315 (PMC6549138; doi:10.26508/lsa.201900315)
Supplement: Supplementary file 1 [file LSA-2019-00315_TableS1.doc]

Supplemental Table S1: Primers used for telomere-PCR, PETRA, hairpin assay and PCR verification of CliP mutants

| Name | Sequence (5'→3') | Description |
| --- | --- | --- |
| oT1090 | ACAAAACCCCTTAAAACCCCCATTTAG | Forward primer for Telomere-PCR |
| 169M | CGGGATCCGGGGGGGGGG | Poly-G Reverse primer for Telomere-PCR |
| oT0958 (CHSB from (Fulneckova et al., 2013) | GTTTTAGGGTTTTAGGGTTTTAGGGTTTTAG | Telomere-specific probe for TRF Southern blot and in-gel hybridization (also called G-probe) |
| oT0959 | CTAAAACCCTAAAACCCTAAAACCCTAAAAC | Telomere-specific probe for in-gel hybridization (also called C-probe). Reverse-complement of oT0958. |
| oT1053 | TGTGTGTGTGTGTGTGTGTGTGTGTG | Used in TRF Southern blot to check for TG microsatellites |
| F_m1_ | AACACATTTGCACACGCAAT | Forward primer used to identify insertion in tel-m1 as suggested in https://www.chlamylibrary.org/feature/LMJ.RY0402.077111_2 |
| R_m1_ | GGAGTGGGGCACAAAGTAGA | Reverse oligo used to identify insertion in tel-m1 as suggested in https://www.chlamylibrary.org/feature/LMJ.RY0402.077111_2 |
| F_m2_ (F12) | TCTGGATCCAAATCCACCGC | Forward primer used to characterize tel-m2 by the absence of a band, present in CC4533- and tel-m1. |
| R_m2_ (R6) | GACCCTGCCACTGCCTTATT | Reverse primer used to characterize tel-m2 by the absence of a band, present in CC4533- and tel-m1. |
| R_m4_ | GGAGATAGCCTGTGAGCCAG | Primer used for the characterization of tel-m4 with primer K_2_. <https://www.chlamylibrary.org/feature/LMJ.RY0402.105594_2> |
| K_1_ (oMJ913) | GCACCAATCATGTCAAGCCT | 5' primer relative to the paromomycin cassette used in the generation of the CliP library (<https://www.chlamylibrary.org/content/Update-how-characterize-insertion-sites-PCR-different-primers-are-needed-mutants-starting>) |
| K_2_ (oMJ944) | GACGTTACAGCACACCCTTG | 3' primer relative to the paromomycin cassette used in the generation of the CliP library (<https://www.chlamylibrary.org/content/Update-how-characterize-insertion-sites-PCR-different-primers-are-needed-mutants-starting>) |
| oT1208 (F_c_; F2) | GCAAACGCTTCATCAGGCAA | Forward primer in the telomerase gene not affected in the WT nor the telomerase mutants |
| oT1209 (R_c_; R2) | GCGTATGACCTACCGGCTAC | Reverse primer in the telomerase gene not affected in the WT nor the telomerase mutants |
| OLIP1 | CCGCACATGAGACGTTACAG | Determination of mating-type + of Chlamydomonas strains. |
| OLIP2 | GATTGCTCTGTCGTTGCAGA | Determination of mating-type + of Chlamydomonas strains. |
| OLIM1 | TGGCGTACCTTTCTGTAGGG | Determination of mating-type - of Chlamydomonas strains. |
| OLIM2 | GCCACG AAG GCAGTTACATT | Determination of mating-type - of Chlamydomonas strains. |
| Blunt Hairpin | GGATCCGACTTTTGTCGGATCC | Used in the hairpin assay. |
| PETRA-T | CTCTAGACTGTGAGACTTGGACTACCCTAAAACCCT | Used in the PETRA experiment. |
| PETRA-A | CTCTAGACTGTGAGACTTGGACTAC | Used in the PETRA experiment. |
| 1R(a) | TACTTGTGTGTGCTGTGCGT | Used in the PETRA experiment for chromosome 1R. |
| 9R(a) | ACAGCACAATACAGTATATA | Used in the PETRA experiment for chromosome 9R. |
| 10R(c) | AACGTCCTCGTGAGACCACC | Used in the PETRA experiment for chromosome 10R. |
| Cr18S_PR1_F | CTTCACTGTCTGGGACTCGGA | Forward primer to amplify a fragment in 18S ribosomal subunit gene from genomic DNA of strain CC4350 |
| Cr18S_PR1_R | ACTAAGAACGGCCATGCACCA | Reverse primer to amplify a fragment in 18S ribosomal subunit gene from genomic DNA of strain CC4350 |
